# Scented Tartary Buckwheat Tea: Aroma Components and Antioxidant Activity

**DOI:** 10.3390/molecules24234368

**Published:** 2019-11-29

**Authors:** Qinglian Xu, Li Wang, Wenxiu Li, Yage Xing, Ping Zhang, Qin Wang, He Li, Hong Liu, Hua Yang, Xiaocui Liu, Yuan Ma

**Affiliations:** 1 Key Laboratory of Grain and Oil Processing and Food Safety of Sichuan Province, College of Food and Bioengineering, Xihua University, Chengdu 610039, China; xuqinglian01@163.com (Q.X.); WANG905359096@163.com (L.W.); 18408248463@163.com (W.L.); lihe@mail.xhu.edu.cn (H.L.); gyliuhong@126.com (H.L.); yang1hua1@yeah.net (H.Y.); xiaocuiliu777@126.com (X.L.); ymxhu@mail.xhu.edu.cn (Y.M.); 2 Huantai Biotechnology Co., Ltd., Chengdu 610225, China; htzp2019@163.com; 3 Department of Nutrition and Food Science, Maryland University, College Park, MD 20742, USA; wangqin@umd.edu

**Keywords:** buckwheat scented tea, aroma components, antioxidant activity

## Abstract

In this study, the aroma compounds of Huantai tartary buckwheat tea (TBH), three laboratory-produced scented tartary buckwheat teas, as well as the antioxidant activity of tea infusion was investigated. In total, 103 aroma components were isolated and identified from all samples. Tartary buckwheat rose tea (TBR) contained 57 aroma components and tartary buckwheat jasmine tea (TBJ) had 53, both of which were higher than those in others. In addition, the total flavonoid content (TFC) and the total phenolic content (TPC) of scented tartary buckwheat tea were much higher than those of TBH. After the tartary buckwheat tea (TBT) was soaked in hot water twice, the antioxidant activity of all samples decreased, and the antioxidant activity of TBR and TBJ infusions was more stable than those of others. Further, the antioxidant activity of the first tea infusion (FTI) of the TBT was higher than that of the second tea infusion (STI). Overall, considering the diverse aroma compounds of scented tartary buckwheat tea and higher antioxidant activity of tea infusions, the combination of scented tea and tartary buckwheat is a feasible approach to develop tartary buckwheat scented tea.

## 1. Introduction

Buckwheat, belonging to the Polygonaceae family [1], is a gluten-free pseudo-cereal, which is cultivated worldwide due to its exceptional adaptability to harsh environments. Many different buckwheat varieties are distributed throughout the world. However, only two species are widely cultivated for human consumption: common buckwheat (*Fagopyrum esculentum Mӧench*) and tartary buckwheat (*Fagopyrum tataricum Gaertn*) [2,3,4]. Common buckwheat was grown extensively in China during the 10th and 13th centuries, was introduced to Europe through Turkey and Russia during the 14th to 15th centuries, and was taken to Great Britain and the United States during the 17th century [5]. Furthermore, buckwheat is a staple food for many rural communities in countries such as Nepal, Bhutan, and China [6]. China is the only country of origin and has the largest area planted with tartary buckwheat in the world. Liangshan Prefecture is the most concentrated and planted area of tartary buckwheat in China and has a long history of cultivation. By 2015, the cultivation area of tartary buckwheat exceeded 370 square kilometers, while more than 55 million kilograms were produced in Liangshan Prefecture. Bitter buckwheat crops are essential sources of income for local farmers. In recent years, an emphasis on the management of diabetes mellitus through dietary solutions has been made in response to an increasing prevalence. Compared to common buckwheat, tartary buckwheat is attracting increased attention due to its unique chemical composition and nutritional efficacy in the prevention and treatment of various diseases. However, there may be some adulteration of tartary buckwheat products due to the lower cost of common buckwheat. Therefore, it is particularly important to use salicylaldehyde, a common aroma compound of common buckwheat, as a marker to detect the contamination/adulteration of tartary buckwheat.

Buckwheat grain is used in various forms for human consumption, as well as in livestock and poultry feed [7]. Whole seeds, obtained after dehulling when raw or following hydrothermal pretreatment (so-called buckwheat groats), can be used similarly to rice, while buckwheat flour can be added to bread dough or pasta products [8]. The flavonoids found in buckwheat have exhibited considerable health benefits, such as hypocholesterolemic, hypoglycemic, and antibacterial effects [9,10]. The content and composition of flavonoids in buckwheat species is diverse. Generally, the content of flavonoids in tartary buckwheat (40 mg/g) was higher than that in common buckwheat (10 mg/g), and reached 100 mg/g in tartary buckwheat flowers, leaves, and stems [11]. More importantly, the nutritional and functional components of buckwheat have been perfectly blended and processed into various foods, such as noodles, bread, pancakes, vinegar, and tea [12,13,14]. In recent years, emphasis on the management of diabetes mellitus through dietary solutions has been made in response to increasing prevalence.

Tartary buckwheat tea (TBT) is not traditional tea (such as black tea, green tea, scented tea, etc.), strictly speaking, it is a kind of roasted rice tea, which is processed from tartary buckwheat seeds (also known as tartary buckwheat rice) after grounding, mixing with water, extruding, dehydrating, drying, and roasting. TBT is increasingly popular in Asia and Europe due to its unique malty aroma, health effects, and convenience. Furthermore, various forms of TBT products are available in both traditional and online markets [9]. However, TBT products in the market are too monotonous, lack novelty, and fail to meet the current needs of consumers. To improve the sensory quality of TBT and enhance its nutritional value, combinations of tartary buckwheat grain with other scented teas were created. Chrysanthemums, roses, and jasmine are widely consumed as scented tea and have been enjoyed for several centuries in China due to their unique aroma and bitter taste [15,16,17]. Based on the differences of raw materials and processing methods, TBT can be generally divided into whole grain tea and whole plant tea. In this study, the nutritional value of TBT was balanced and diversified by mixing and granulating tartary buckwheat grain and scented tea in the proper proportions. More importantly, the scented tea could be processed simply at low cost containing environmentally friendly properties. The scorching and bitter flavor of tartary buckwheat would be concealed by scented tea, improving the taste and eventually increasing its market value. 

Consumers favor TBT due to its unique flavor and excellent health benefits. However, although extensive research has been conducted into the volatile aroma composition and antioxidant activity of TBT [12,18,19], few studies were performed to detect the aroma components of scented tartary buckwheat tea and the antioxidant properties of tea infusion. Therefore, the aroma components of Huantai tartary buckwheat tea (TBH, a marketed TBT) and three laboratory-produced buckwheat scented teas were identified using an electronic nose (E-nose) and headspace solid-phase microextraction-gas chromatography-mass spectrometry (HS-SPME-GC-MS) in this experiment. The antioxidant activity of tea did not reflect the intake of the human body, because the body absorbed the tea soup instead of tea. Therefore, the total flavonoid content (TFC), total phenolic content (TPC), DPPH free radical scavenging rate, total reducing capacity, and hydroxyl radical scavenging ability during the first tea infusion (FTI) and second tea infusion (STI) were determined simultaneously.

## 2. Results and Discussion

### 2.1. E-Nose Analysis

**Figure 1:**
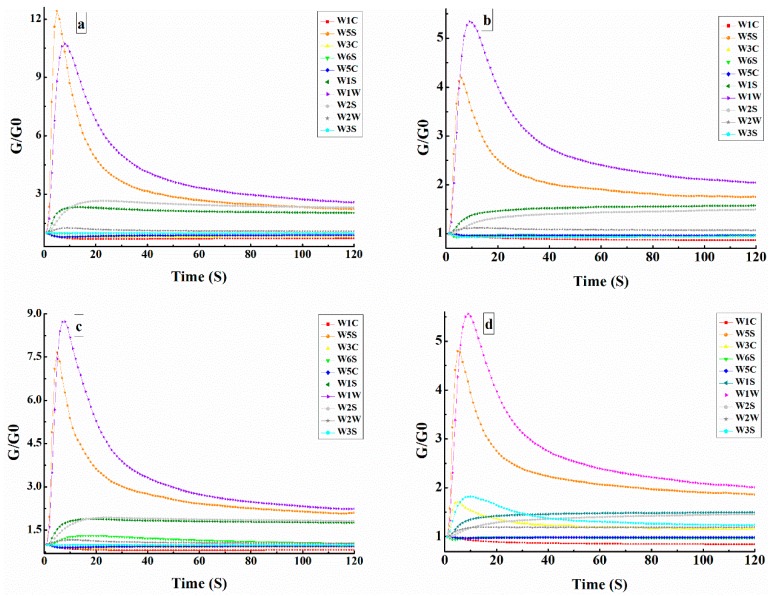
A typical response of ten sensors during the measurement of TBH (**a**), TBC (**b**), TBR (**c**) and TBJ (**d**). (Abbreviations: TBH—Huantai tartary buckwheat tea, TBC—tartary buckwheat chrysanthemum tea, TBR—tartary buckwheat rose tea, TBJ—tartary buckwheat jasmine tea).

The response value of the E-nose sensors was expressed by the conductivity ratio (G/G0), where G and G0 were the conductivity of the sensors when the sample gas and reference air flowed over the measurement chamber, respectively [20]. Figure 1a–d depicts the typical sensor responses for TBT. Each curve represents the response value of each sensor to time during the measurement. In this study, the response value of the sensor stabilized within 120 s and was used for future analysis. Following a low and stable resistance during an initial period (0–12 s), the conductivities of sensors W1W, W5S, W1S, and W2S increased sharply and then remained stable to the end (120 s). In addition, almost no changes occurred for the other sensors during the detection time, particularly W1C, W6S, and W5C.

In this study, the relative levels of W5S, W1S, W2S, and W1W for TBT displayed a visible increase, while the relative levels of W3C, W6S, W5C, W2W, W3S, and W1C remained almost stationary during the entire test period. These results indicated that various chemical substances such as terpenes, aromatic hydrocarbons, and alcohols were formed during the tea-making process. Yang and others used the E-nose to evaluate the aromas of non-fermented Pu-erh tea from ten different storage years (2006 to 2015). W1W, W2W, W1S, and W5C were the most sensitive of the ten sensors used to detect the aroma of dry tea leaves, tea infusions, and infused leaves [21]. Lan found that W1W, W5S, W2S, and W1S were the most sensitive to wine samples using the E-nose to determine the complete flavor profile of pomegranate wines [22]. The E-nose is sensitive for obtaining odor information from samples, and slight changes in volatile compounds may cause a difference in the response of the sensors [23].

#### 2.1.1. Principal Component Analysis (PCA) of the Volatile Compounds in Tartary Buckwheat Tea (TBT)

**Figure 2:**
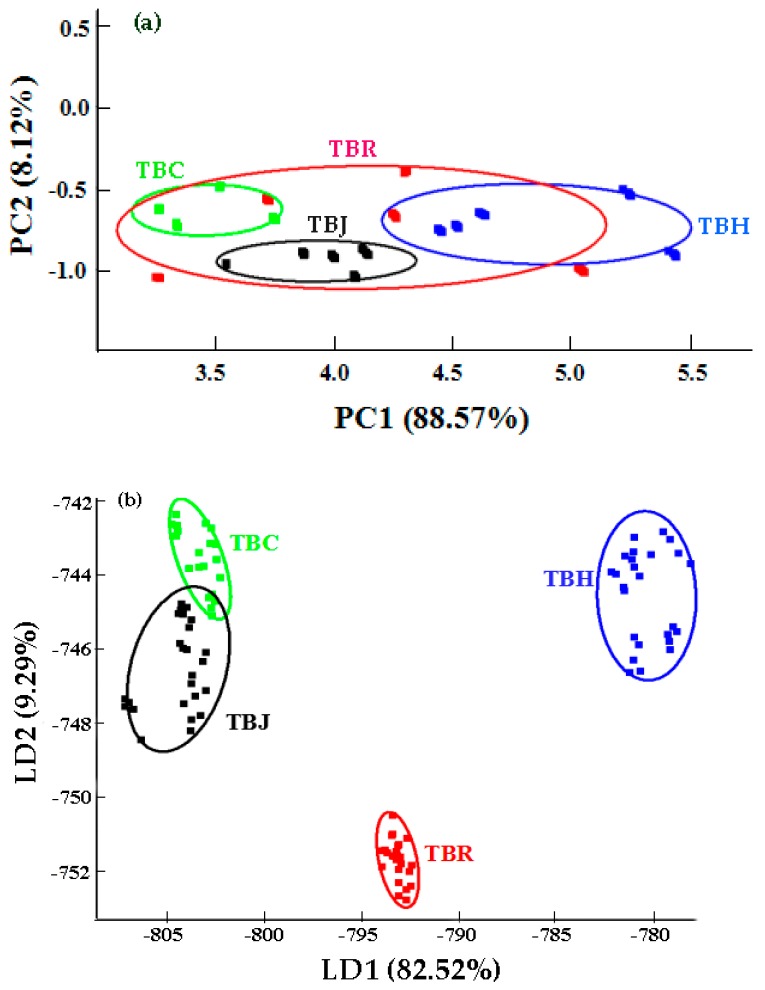
Principal component analysis (**a**) and Linear discriminant analysis (**b**) of E-nose data for TBT. (Abbreviations: TBH—Huantai tartary buckwheat tea, TBC—tartary buckwheat chrysanthemum tea, TBR—tartary buckwheat rose tea, TBJ—tartary buckwheat jasmine tea).

PCA was used to analyze the response values of the sensors. In Figure 2a, each circle represents a sample, and the distance between any two points represents the extent of the differences between those samples [24]. The contribution rate of the first principal component was 88.57%, and that of the second principal component was 8.12%. Since the total contribution rate explained 96.69% of the total variation, the first two principal components were selected. Furthermore, the cumulative contribution rate of the two principal components was more than 96%, indicating that the two principal components represented the main information characteristics of the sample, and could be used to analyze the correlation of volatile components in different kinds of TBT. A partial overlap was evident between tartary buckwheat rose tea (TBR) and the other three samples, indicating that they possessed similar volatile components, while distinct differences existed between TBH, tartary buckwheat chrysanthemum tea (TBC), and tartary buckwheat jasmine tea (TBJ) with no overlap among them.

PCA is an unsupervised method that reduces multidimensional data to orthogonal coordinates based on maximum variance linear projection and is used to convert the data into two-dimensional (2D) or three-dimensional (3D) coordinates. In the 2D and 3D PCA plots, samples with similar patterns are placed together, and the different groups can be visually displayed [20]. In this study, PCA was used to derive the first two principal components from the E-nose data and to visualize the information that exists in the data [20]. Furthermore, 2D or 3D coordinates are constructed by the top two or three PCs, and their corresponding score plot shows the relation between the observations [25]. Additionally, PC1 and PC2 were used to construct a 2D scatter plot to visualize the cluster trend of these samples. Moreover, Lan et al. [22] used PCA to analyze the volatile compounds in pomegranate wine at different fermentation times. After reducing the dimension from ten variables to two principal components, the value of PC1 increased, while the value of PC2 displayed no significant change during the entire winemaking process. Considering the volatile components of the wine before fermentation, similar levels were evident on the fourth day of the fermentation process, while significant differences were detected on day 26.

#### 2.1.2. Linear Discriminant Analysis (LDA) of the Volatile Compounds in Tartary Buckwheat Tea (TBT)

LDA can project a high-dimension pattern to identify the vector space best and extract classification information to reduce the required dimension. Additionally, this reduction makes it more open, improving sample differentiation in the presence of a larger LDA value [21]. The chart of LDA discrimination factor analysis is shown in Figure 2b. TBH and TBR were obviously separated but the other samples overlapped to a certain extent. The contribution rate of discriminant LD1 and LD2 was 82.52% and 9.29%, respectively, and the total contribution rate was 91.81%. Therefore, different volatile components could be adequately detected and distinguished by the E-nose, and the large distance between TBH and three kinds of scented tartary buckwheat tea was clearly observed. In addition, it is significantly different from other samples on the LD1 axis, which may be caused by the fact that TBH has special volatile compounds different from other samples and can be recognized in LDA. In this experiment, the LDA results for TBT exceeded those obtained from the PCA analysis. Moreover, LDA technology could reduce the drift effect observed in the response of the E-nose, thus improving the classification accuracy.

### 2.2. Identification of the Aroma Compounds in Tartary Buckwheat Tea (TBT)

**Figure 3:**
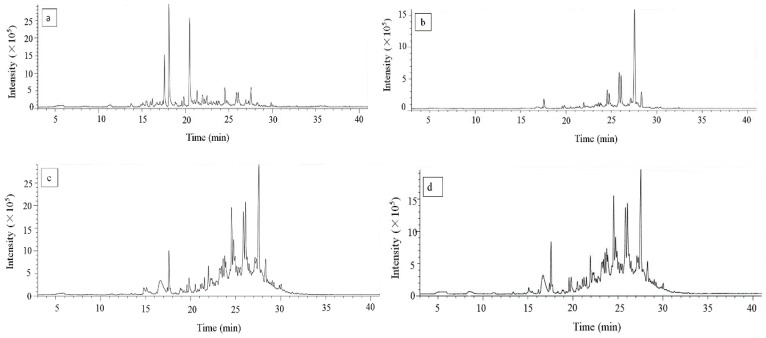
GC-MS total ion chromatograms of aroma components of TBH (**a**), TBC (**b**), TBR (**c**) and TBJ (**d**) (Abbreviations: TBH—Huantai tartary buckwheat tea, TBC—tartary buckwheat chrysanthemum tea, TBR—tartary buckwheat rose tea, TBJ—tartary buckwheat jasmine tea).

The SPME technique has many advantages, and is used widely in the fields of medicine, food, agriculture, as well as the environment. Furthermore, there is no need for large amounts of organic solvent, while the operation is convenient and accurate [18]. In this study, the aroma components in TBT samples exhibiting special malt flavor were identified with GC-MS. Typical chromatograms depicting the total ion levels of the aroma compounds extracted with SPME are shown in Figure 3a–d. Only the results of quality >80% are listed in Table 1. Furthermore, 103 volatile substances, including eight esters, 16 alcohols, six aldehydes, eight ketones, 56 hydrocarbons, and nine others were identified in the samples. Among these compounds, 17 were also found in other reports [9,18,19]. Eight of these compounds, namely nonanal, decanal, l-caryophyllene, farnesene, undecane, dodecane, 2,6,10-trimethyldodecane, and 3, 8-dimethyldecane, were detected in all the samples. The relative ester content in TBH was higher than that in other samples, while the relative content of dimethyl glutarate and dimethyl adipate were 16.60% and 17.01%, respectively. However, the relative content of alloaromadendrene was the highest in TBC, TBR, and TBJ, at 30.37%, 13.68%, and 10.67%, respectively.

**Figure 4:**
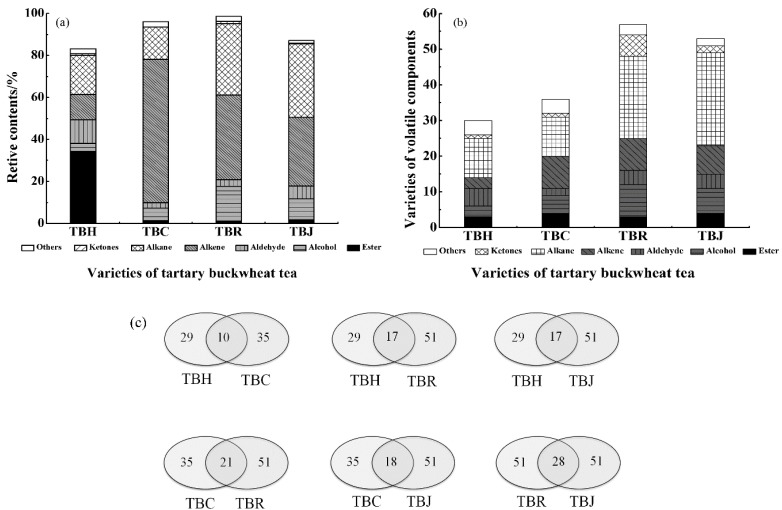
Comparison of volatile components content in tartary buckwheat tea (TBT) (**a**); Varieties of volatile components in TBT (**b**); Analysis of volatile components common to TBT (**c**). (Abbreviations: TBH—Huantai tartary buckwheat tea, TBC—tartary buckwheat chrysanthemum tea, TBR—tartary buckwheat rose tea, TBJ—tartary buckwheat jasmine tea).

A comparison of volatile component content and varieties of aroma components in TBT varied obviously, as shown in Figure 4a,b. The esters accounted for a large proportion in TBH (34.35%), while higher alkene levels were evident in TBC (68.29%), TBR (40.26%), and TBJ (32.78%). Moreover, besides the alkane compounds numbering 23 and 26, respectively in TBR and TBJ, 51 aroma component species were also identified, exceeding those detected in other samples, while several distinctly different chemical components were found in TBT. A high level of a unique dimethyl adipate was found in TBH, while TBR contained higher levels of alloaromadendrene. TBJ had more species but a lower relative content of aroma components. Moreover, the relative content of esters in TBH was the highest (34.35%), accounting for 41.29% of the total. The relative contents of the alkene in TBC and TBR were the highest at 68.29% and 40.26%, respectively, accounting for 70.86% and 40.85% of all components. However, the proportion of olefins and alkanes in TBJ were essentially equal at 32.78% and 34.87%, respectively, which was more than 67.65% of all components.

The results derived from the statistical analysis of the common volatile compounds in all samples are shown in Figure 4c. TBH and TBR shared 17 common components, as did TBH and TBJ. Furthermore, 21 shared substances were found in TBC and TBJ, while TBR and TBJ contained 28, showing a high similarity in volatile components. These results were consistent with those obtained from the PCA analysis of the E-nose. Therefore, it is evident that combining the E-nose with PCA and LDA analysis, as well as GC-MS, could successfully distinguish between different types of TBT.

Besides their exceptional nutritional value, buckwheat products possess a distinct aroma. The aroma is highly valued by consumers and represents the most significant feature denoting the quality of TBT and can be attributed partly to the basic materials used and partly to the production process. Therefore, consumers prefer TBT due to its unique flavor and beneficial health effects. After performing three different separation techniques, Qin et al. [19] analyzed the aroma compounds of TBT using GC-MS, identifying 77 compounds of which 35 were quantified according to the available standards. The compounds that contributed significantly to the aroma of TBT included 2,5-dimethyl-4-hydroxy-3(2*H*)-furanone, nonanal, 2,3-diethyl-5-methylpyrazine, benzeneacetaldehyde, maltol, 2,5-dimethylpyrazine, 2-ethyl-5-methylpyrazine, and trimethylpyrazine. Moreover, several nutritional and bioactive compounds, such as linoleic acid, niacin, vanillic acid, 7-hydroxycoumarin, and butylated hydroxytoluene, were determined in this study. In addition, further research determined the volatile components of two kinds of TBT, whole grain tea and whole plant tea [9]. GC-MS analysis identified 14 aroma components in whole grain tea and whole plant tea, with 3-ethyl 2,5-dimethylpyrazine as the main component. However, some differences were evident in the aroma composition profile. Nevertheless, the relative content of these compounds varied among the different types of TBT, indicating that the raw materials affected the content of aroma compounds significantly, resulting in odor differences in the tea [18]. Moreover, aroma compounds were different in different kinds of TBT, indicating that the raw material was one of the important factors affecting the odor of the product. The salicylaldehyde content in buckwheat groats and flour samples was determined by Janeš. Results showed that traditionally dehulled buckwheat grain, which had the strongest odor, contained the highest concentration (1.6 ppm) of salicylaldehyde with an odor activity value (OAV) of 216, which indicated that salicylaldehyde was the most characteristic compound of common buckwheat [18]. Starowicz also reported that the most important difference of the aroma of tartary buckwheat was the absence of salicylaldehyde, indicating the potential to use salicylaldehyde as a marker to detect the contamination/adulteration of tartary buckwheat with common buckwheat or vice versa [26]. Salicylaldehyde was not found in all TBT analyzed in this study.

### 2.3. Total Flavonoid Content (TFC) and Total Phenolic Content (TPC) in Tartary Buckwheat Tea (TBT)

**Figure 5:**
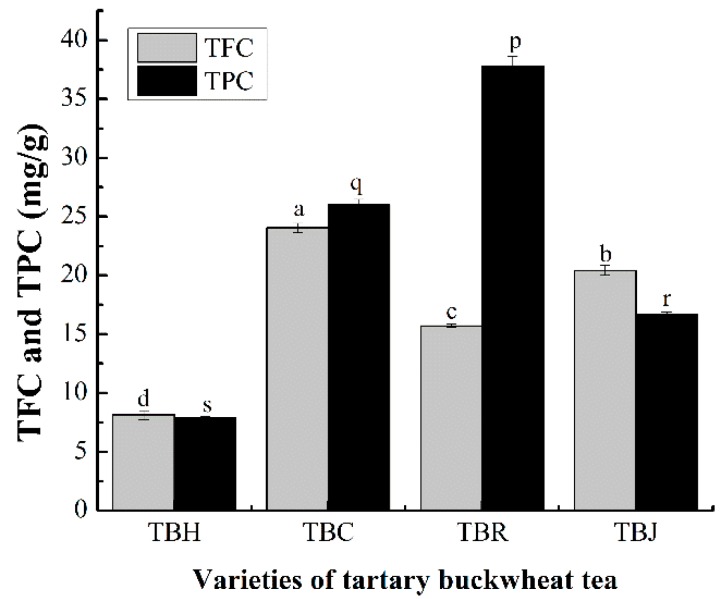
The total flavonoid content (TFC) and total phenolic content (TPC) in tartary buckwheat tea (TBT) (The same letter in the Figures signifies no significant difference, and the letters a–d and p–s indicates the difference of the total flavonoid content (TFC) and total phenolic content (TPC), respectively (*p* < 0.05)).

Flavonoids are an important bioactive component in TBT. To identify the profile of flavonoids in TBT, the TFC in TBH and scented tartary buckwheat tea were determined. As shown in Figure 5, the TFC of the TBT ranged from 8.12 ± 0.38 mg/g to 24.04 ± 0.37 mg/g with significant differences (*p* < 0.05), and the TFC in scented tartary buckwheat tea was higher than that in TBH. Obviously, the TFC of the TBC was the highest (24.04 ± 0.37 mg/g), and the TFC of TBH was the lowest (8.12 ± 0.38 mg/g), the former was about three times as much as the latter. The total phenols in all samples were also determined. As shown in Figure 5, there was an apparent difference in the TPC of four species of TBT on average. Further, the TPC in alcohol extracts of TBH was much lower than that of scented tartary buckwheat tea. The TPC of TBR was the highest (7.88 ± 0.09 mg/g) and that of TBH was the lowest (37.78 ± 0.82 mg/g). It was reported that the TPC in hulls and bran of common buckwheat and tartary buckwheat were higher than in the flour, while the TPC of tartary buckwheat was higher than that in common buckwheat.

Qin [27] collected 39 buckwheat cultivars from China to investigate the nutritional composition and flavonoids content of flour from different buckwheat cultivars, including 21 tartary buckwheat and 18 common buckwheat. This study found that the contents of proteins, fats, and crude fibers of the tartary buckwheat flour were similar to those of common buckwheat flour, while the flour of common buckwheat showed a low level of flavonoids, indicating that the chemical properties of buckwheat and its products may be affected by different processing methods and raw materials. Liu et al. [28] found that tartary buckwheat has a higher level of TPC and TFC compared to common buckwheat. All thermal treatments particularly microwave cooking, contributed to the greatest losses of TPC and antioxidant capacities in the common buckwheat while boiling and steaming usually lost the least. For the tartary buckwheat samples, all thermal treatments (except roasting), especially boiling and steaming, led to significant increases in TPC and TFC. Therefore, processing mode is one of the important factors affecting product quality.

### 2.4. Total Flavonoid Content (TFC) and Total Phenolic Content (TPC) in the Tea Infusion of Tartary Buckwheat Tea (TBT)

#### 2.4.1. Total Flavonoid Content (TFC) in the Tea Infusion of Tartary Buckwheat Tea (TBT)

Buckwheat, which is rich in flavonoids, is the main source of rutin in the human diet. As shown in Table 2, the TFC of the tea infusion was determined spectrophotometrically, and ranged from 3.33 ± 0.27 mg/g to 12.03 ± 0.87 mg/g with significant differences (*p* < 0.05). The TFC levels of the TBC infusions were highest in the FTI at 12.03 ± 0.87 mg/g, while the STI value was 8.98 ± 0.84 mg/g. Following two brewing cycles, the TFC of the TBH infusion decreased by 34.34%, while that of TBR displayed no significant change with values of 3.73 ± 0.12 mg/g in the FTI and 3.58 ± 0.26 mg/g in the STI. Furthermore, the flavonoids of TBR exhibited the highest thermal stability of all the samples, while the opposite was apparent in TBH.

Flavonoids are the most common and widely distributed phenolic compounds in plants [29]. Guo et al. [9] reported that the flavonoid content dissolved in hot water displayed substantially lower levels than that of the TBT alcohol extracts, and the TFC determined in the tea infusion exhibited a 60% decline compared with that of the TBH alcohol extracts. Moreover, the TFC in the FTI of whole grain tea was 4.66 ± 0.03 mg/g, and whole plant tea was 0.58 ± 0.01 mg/g, which was 7.04 ± 0.01 mg/g in this study. Early research suggested that tartary buckwheat seeds contained rutin in the range of 800 mg to 1700 mg rutin/100 g DW, with only trace amounts of quercetin, the aglycone of rutin [30]. This difference may be due to genetic variations, maturity, and environmental factors such as sun exposure, rainfall, and different cultivation types [31]. The results indicate the potential of tartary buckwheat as a functional ingredient with excellent application prospects.

#### 2.4.2. Total Phenolic Content (TPC) in the Tea Infusion of Tartary Buckwheat Tea (TBT)

Phenolic compounds are bioactive components found in plant products and possess significant health benefits, while playing an important role in the antioxidant activity of plant materials [32]. As shown in Table 2, the TPC of TBT varied with the species and brewing time, while a significant difference existed in the TPC of both the FTI and STI (*p* < 0.05). After repeating the brewing process twice, the TPC of all samples decreased, falling by 19.80% in TBH, from 5.17 ± 0.06 mg/g in the FTI to 4.14 ± 0.01 mg/g in the STI. Furthermore, the TPC in the tea infusion of scented tartary buckwheat tea (4.58 ± 0.02 mg/g–9.49± 0.01 mg/g) was much higher than that of TBH (4.14 ± 0.01 mg/g–5.17 ± 0.06 mg/g) in this study. This was consistent with the above mentioned TFC and TPC of scented tartary buckwheat tea were both higher than TBH. The TPC of the TBR infusion declined slightly, from 9.49± 0.01 mg/g (in the FTI) to 9.27± 0.09 mg/g (in the STI), while a considerable reduction of 19.80% was observed in TBH (from 5.17 ± 0.06 mg/g to 4.14± 0.01 mg/g). Following a brewing process that was repeated twice, the results showed that the total phenols of TBR displayed the highest content and the best stability.

Polyphenolic compounds belong to a heterogeneous group, which display a substantial variety of beneficial biological effects, including anti-inflammatory, anti-microbial and antioxidant properties [33]. It was reported that the TPC in common and tartary buckwheat hulls and bran were higher than in the flour, while the TPC of tartary buckwheat bran and flour were higher than in common buckwheat [20]. Moreover, Zielinska et al. [34] prepared tea from the buckwheat hulls to examine its TPC, TFC, antioxidant capacity, and anti-glycation activity. Results indicated that the number of phenolic compounds extracted with 80% methanol from dry buckwheat hulls (3.56 ± 0.19 mg/g) was 64 times lower than that of green tea leaves. In addition, water temperature and soaking time may also affect the TPC of infusion [35].

### 2.5. Antioxidant Ability of the Tea Infusion of Tartary Buckwheat Tea (TBT)

Antioxidant activity is crucial in reflecting the biological activity of tea infusions. Furthermore, TPC and TFC do not necessarily indicate the antioxidant activity in plants and, therefore, the antioxidant activity of each phenolic compound should be considered quantitatively [1]. DPPH, a stable radical, is widely used to assess the free radical scavenging ability of various samples. The hydroxyl radical scavenging activity assay is an essential tool for determining antioxidant capacity [36]. In this study, the DPPH, •OH free radical scavenging ability, and total reducing power were determined to measure the antioxidant capacity of tea infusions by examining eight specific samples (Table 2). TBT was soaked twice in hot water, and the DPPH radical scavenging activity, •OH radical scavenging ability, and total reducing power of the STI was reported in the following order: TBC > TBJ > TBR > TBH, TBJ > TBR > TBC > TBH, TBR > TBC > TBJ > TBH. Among the extracts, the DPPH free radical scavenging ability appeared to be the lowest in TBH when compared with other TBT. Furthermore, the free radical scavenging rate of DPPH in TBJ decreased clearly, from 76.30 ± 1.19% in the FTI to 62.33 ± 5.71% in the STI. The •OH radical scavenging ability of the TBH and TBC infusions exhibited a visible decline of 36.07% and 32.35%, respectively. Additionally, the results indicated that the total reducing power of the TBT infusion declined in conjunction with an increase in the brewing times. Furthermore, the total reducing power in TBH decreased rapidly from 1.81 ± 0.01 in the FTI to 1.32 ± 0.04 in the STI and displayed a slight reduction in both TBC (9.33%) and TBR (5.13%). Therefore, in this research it was found that the highest levels and most stable antioxidant activity in the tea infusions of all the samples, presented in the following order: TBR > TBJ > TBC > TBH. Results indicated the following order to denote the stability of the DPPH and •OH radical scavenging ability, as well as the total reducing power in the tea infusion of TBT soaked in hot water twice: TBC > TBR > TBH > TBJ, TBJ > TBR > TBC > TBH and TBR > TBC > TBJ > TBH, respectively.

Free radicals are irreplaceable in the origin of life and in the process of biological evolution, suggesting their beneficial effects on the organisms. The health benefits of bioactive compounds, such as the anti-carcinogenic and anti-inflammatory effects, depend on their antioxidative capacity. Antioxidant activity is crucial in reflecting the biological activity of tea infusions [37]. In addition, two or more methods regarding the mechanism of scavenging free radicals and reducing power are usually selected to evaluate the antioxidative activity of samples, considering that the determination of the capacity varies according to their antioxidative mechanisms [38]; the use of multiple assays is, therefore, preferable [39]. Additionally, the antioxidant activity of tartary buckwheat is not only affected by processing methods, but also by the specific part of the plant that is used. Lee and others found that tartary groats contained the highest levels of phenolic compounds and displayed the highest antioxidant activity. In particular, the antioxidant activity of tartary groats is three to five times higher than that of common groats, while the rutin content is 70 times higher indicating that tartary buckwheat can be used as a vital antioxidant in food [39]. Additionally, the antioxidant activity of buckwheat extract was affected by the extraction solvent, as well as the analysis method [40].

## 3. Materials and Methods

### 3.1. Materials and Chemicals

TBH and tartary buckwheat grain (*Chuanqiao NO.2*) were obtained from Huantai Biotechnology Co., Ltd. (Liangshan Prefecture, China). Chrysanthemums, roses, and jasmine were obtained from Jin Shanghao Tea Co., Ltd. (Xiamen, China). Gallic acid, DPPH, catechol and rutin were purchased from Tixi Chemical Industrial Development Co. Other chemicals and reagents were purchased locally and were of analytical grade.

### 3.2. Preparation of the Samples

The three types of laboratory-produced buckwheat scented teas included TBC, TBR and TBJ. The tartary buckwheat was subjected to a process that included washing, drying, and pulverization, after which it was mixed with the scented tea in equal proportions, followed by the addition of water to allow for granulation. After pelletizing and shaping, it was dried and baked, and the tartary buckwheat compound tea was obtained after cooling. All samples were dried at room temperature and stored in a dry, cool place for later use.

#### 3.2.1. Preparation of the Tea Powder

The samples were dried in a constant temperature blast oven at 60 °C for 1 h, crushed to 150 μm with a high-speed pulverizer, and prepared for E-nose and GC-MS analysis.

#### 3.2.2. Extraction of Total Flavonoids and Total Phenols

Using an ultrasonic device, 1.0 g of tea powder was extracted with 55 mL of 75% ethanol at 45 °C for 75 min. The solution was filtrated and made up to a total volume of 50 mL using 75% ethanol for subsequent quantitative analyses of total flavonoids and total phenols.

#### 3.2.3. Preparation of the Tea Infusion

The tea infusion was prepared as follows: 10 g of tea was extracted with 100 mL water at 90 °C by an ultrasonic device for 10 min. Then, the water extract was filtered with a 0.22 mm nylon filter membrane (Jin Teng Experiment Equipment Co., Ltd., Tianjin, China) after cooling to room temperature. The filtrate was the first tea infusion (FTI) and the above procedure was repeated to get the second tea infusion (STI). All samples were stored at 4 °C before use.

### 3.3. E-Nose Analysis

The emission of volatile compounds was monitored using a commercial portable E-nose. The Portable Electronic Nose 3.0 (PEN3) from AIRSENSE Analytics (Schwerin, Germany) has an array of ten different metal oxide sensors. The overall flavor of all samples was determined by Torri’s procedure with slight modifications, and five replications were performed for each sample [41]. During the E-nose analysis, 3 g tea powder was placed in a 50 mL airtight glass vial and sealed with a polytetrafluoroethylene (PTFE) bolt and nut. Following the equilibrium process at 60 °C for 30 min, the glass bottles containing the samples were analyzed at room temperature under standardized conditions. Furthermore, the measurement device absorbed the gaseous compounds from the headspace bottles through the sensor array at a rate of 300 mL/min for 120 s. Then, filtered air was used to purify the system at a flow rate of 600 mL/min for 80 s to allow the instrument to re-establish a baseline for the next sample injection.

### 3.4. GC-MS Analysis of the Volatile Flavor Compounds

The aroma compounds of all samples were extracted using HS-SPME-GC-MS following Bradford’s procedure [18], while Divinylbenzene/Polydimethylsiloxane (DVB/PDMS) SPME 65 μm fiber was used (Supelco, PA, USA). Initially, 1 g tea powder was placed into a 15 mL glass bottle, crimped, and maintained at 40 °C for 30 min. Then, the SPME fiber was inserted into the headspace where the position was maintained for 60 min. Finally, the fiber was placed into the injector port of a gas chromatograph to desorb for 5 min at 250 °C. 

Gas chromatography-mass spectrometry (GC/MS-QP2010; Shimadzu, Kyoto, Japan) was used to test the samples. The aroma compounds were separated with a DB-5 MS capillary column (30 m × 0.25 mm × 0.25 μm) from Agilent, CA, USA. The temperature procedure was performed as follows: the starting temperature was set to 40 °C and maintained for 3 min, then increased to 150 °C at a rate of 5 °C/min where it was maintained for 2 min. The temperature was further increased to 210 °C at a rate of 10 °C/min where it was held for 2 min. The carrier gas (He) flow rate was 1.2 mL/min, and the split ratio was 5:1, while the injection volume was 1 μL. The mass spectrum conditions were as follows: electron bombardment ion source, total ion current (TIC) recorded, electron energy 70 eV, ion source temperature at 210 °C, and interface temperature at 230 °C.

A mass spectral library (NIST11 Database, Agilent Technologies) was used to verify the compound identity when the matching degree was greater than 80%. The relative content of aroma components was expressed by the ratio of the peak area to the total peak area of each aroma component.

### 3.5. Determination of the Total Flavonoid Content (TFC)

The TFC of the samples were determined with the aluminum chloride colorimetric method [29,42] with slight modifications. Briefly, 1.0 mL of the tea infusion was mixed with 4.0 mL of 60% ethanol and 0.3 mL of 5% sodium nitrate (NaNO_2_). After 6 min, 0.3 mL of 10% aluminum nitrate (Al(NO_3_)_3_) was added and left to stand for another 6 min before 4.0 mL of sodium hydroxide solution was added. Then, deionized water was used to adjust the volume to 10.0 mL. Following incubation at room temperature for 15 min, the absorbance of the reaction mixture was measured at 510 nm using an enzyme-labeling measuring instrument (SpectraMax-i3x, Shanghai, China), and compared to that of the rutin standards. With different concentrations of rutin (0–0.5 mg/mL, containing 60% ethanol) as the standard, a standard curve was drawn, and the linear regression equation was given as: Absorbance = 8.520 rutin (mg/mL) + 0.009 (R^2^ = 0.994)). Samples were analyzed in triplicate.

### 3.6. Determination of the Total Phenolic Content (TPC)

The TPC of all samples was determined using the Folin-Ciocalteau reagent [43] with slight modifications. Briefly, 0.6 mL of the tea infusion was mixed with 3 mL of the Folin-Ciocalteau reagent and 1.4 mL water. After 5 min, 2.4 mL of 0.7 M sodium carbonate solution was added, and the mixture was incubated for 2 h in the dark at room temperature. This process was followed by transferring 200 µL of the mixture from the microtube to a clear 96-well microplate, and the absorbance of each well was read at 760 nm by enzyme-labeling measuring instrument. A standard curve was plotted using gallic acid (0.02–0.2 mg/mL in water) as a standard, providing a linear regression equation as follows: Absorbance = 0.008 gallic acid (μg/mL + 0.075 (R^2^ = 0.997)). Samples were analyzed in triplicate.

### 3.7. Determination of Antioxidant Capacity

#### 3.7.1. DPPH Radical Scavenging Activity Assay

The effect of the extracts on DPPH radical scavenging activity was determined with the modified method [44]. Firstly, 2.0 mL of water extract was mixed with 2.0 mL of 0.1 mM DPPH solution (in anhydrous ethanol) and placed in the dark for 30 min after shaking. Then, the absorbance was measured at 517 nm against the blank (mixture without extract). The inhibition rate (%) of DPPH was calculated using the following equation:

DPPH scavenging activity (%) = (A − B)/A × 100(1)

where A = absorbance of the DPPH solution + anhydrous ethanol and B = absorbance of the DPPH solution + test samples. All measurements were performed in triplicate.

#### 3.7.2. Hydroxyl Radical Scavenging Activity Assay

The hydroxyl radical scavenging activity of the tea infusion was measured with the salicylic acid method described by Li et al. [45] with slight modifications. Briefly, 2 mL of extract was added to 1 mL of 9 mM FeSO_4_ solution and 2 mL of 9 mM salicylic acid-ethanol solution. Then, 2 mL of 8.8 mM H_2_O_2_ was added and incubated immediately at room temperature for 1 h. Finally, the absorbance of the mixture was measured at 510 nm. The following formula was used to calculate the scavenging activity of hydroxyl radicals:

(2) $$\mathrm{Hydroxyl}\;\mathrm{radical}\;\mathrm{scavenging}\;\mathrm{activity}\;\left(\%\right)\;=\;\left(1-\frac{A_{X}-A_{X0}}{A_{0}}\right)\times 100$$

where *A_X_* is the absorbance of the sample, *A*_*X*0_ is the absorbance of the control without H_2_O_2_, and *A*_0_ is the absorbance of the blank without the sample.

#### 3.7.3. Total Reducing Power Assay

The reducing power was assayed as described by Zhai et al. [46] with slight modifications. 2.5 mL of extract was mixed with 2.5 mL of phosphate buffer (0.2 M, pH 6.6) and 2.5 mL of 1% potassium ferricyanide. Then, the mixture was incubated at 50 °C for 20 min before adding 2.5 mL of 10% trichloroacetic acid and centrifuging at 3000 r/min for 10 min. Finally, 2.5 mL of supernatant was mixed with 0.5 mL of FeCl_3_ solution (0.1%, *w*/*v*) and 2 mL of distilled water. The absorbance of the mixture was determined at 700 nm. The higher the absorbance value, the stronger the reducing power.

### 3.8. Statistical Analysis

Pattern recognition software (WinMuster 1.6.2) for data recording and elaboration was used to analyze the E-nose examination results. The antioxidant activity data of the tea infusion were expressed as mean ± standard deviation (SD) for the three replicates of each sample and plotted with Origin 8.5 software. An analysis of significant difference tests was conducted with SPSS 20.0 software (SSPS, Inc., Chicago, IL, USA) using one-way analysis of variance (ANOVA) and Duncan’s multiple range tests, and *p* < 0.05 was deemed significant. The same letter in the figures signifies no significant difference, and the letters p–s and a–d indicate the difference of the FTI and STI, respectively.

## 4. Conclusions

In this experiment, the aroma components of the four kinds of TBT were detected using E-nose and GC-MS. In this experiment, the LDA results for TBT exceeded those obtained from the PCA analysis. Under the optimal experimental conditions, 103 volatile flavor compounds were detected by GC-MS, of which eight ingredients were shared. Furthermore, TBR displayed the highest relative content, as well as the highest number of species, containing alcohols, hydrocarbons, and nitrogen-containing compounds. Esters contributed significantly to the flavor of TBH, while the aroma of TBC and TBJ was mainly derived from alkane compounds. Several chemical components exhibiting distinct differences were identified in various bitter buckwheat teas. Furthermore, the results regarding the examination of the tea infusion indicated that the antioxidant activity of all samples decreased after soaking twice. The antioxidant activity of the TBR and TBJ infusions remained strong and stable, while that of the TBH infusion was exceedingly lower than in other samples. These results may be related to the properties of scented tea, which require further study. Although tartary buckwheat is abundant in China, especially in Liangshan, Sichuan Province, the exploitation and utilization of products derived from this plant remain in its infancy. Therefore, critical exploration is necessary regarding new processing technology and diversified products. The results suggest that scented tea provides a promising product that can add value and remarkably improve the antioxidant activity of TBT for enhanced health benefits.

Most flavonoids are converted from rutin to quercetin during processing, which significantly reduces the levels of dissolved flavonoids in tea infusion. Expectedly, rutin will be retained as the main flavonoid in TBT, while the extraction rate of other flavonoids will be improved. Hydrothermal treatment is proposed as the first step in deactivating rutin-degrading enzymes during processing. Furthermore, technologies for enhancing the solubility of flavonoids in water need further study. More importantly, the beneficial effects of TBT should be evaluated by combining its aroma components and antioxidant properties with its specific tea infusion. Moreover, further research is necessary regarding the optimization of preparation process, analysis of antioxidant activity, and sensory analysis of scented tartary buckwheat tea in an attempt to explain and confirm some of the results obtained in this study.

## Figures and Tables

**Table 1:** Volatile components and relative contents of tartary buckwheat tea (TBT).

No.	Compounds	Relative Contents/%			
		TBH	TBC	TBR	TBJ
1	Dimethyl butanedioate	0.74	-	-	0.05
2	Methyl glutarate	16.60	-	-	-
3	Dimethyl adipate	17.01	-	-	-
4	Octyl methacrylate	-	0.23	-	-
5	Z-3-Hexenyl valerate	-	0.08	0.17	0.20
6	Hexyl pentanoate	-	0.55	0.24	0.31
7	Hexyl isovalerate standard for gc (Butanoic acid,3-methyl-, hexyl ester)	-	0.34	0.47	0.52
8	Propanoicacid,2-methyl-	-	-	-	0.69
9	2-Hexyl-1-decanol	1.72	-	1.80	1.08
10	Perilla alcohol	1.64	3.07	-	-
11	2-Butyl-1-octanol	0.32	-	-	0.67
12	2-Ethyl-4-methylpentan-1-ol	-	0.99	-	-
13	2-Isopropyl-5-methyl-1-heptanol	-	1.06	-	-
14	2-Ethylhexanol	-	0.24	2.29	-
15	Tetrahydrolavandulol	-	0.70	1.87	2.02
16	1-Pentacontanol	-	-	2.07	-
17	1-Dodecanol, 2-hexyl-	-	-	1.94	-
18	2-Ethyl-1-dodecanol	-	-	0.99	-
19	(2,2,6-trimethylbicyclo [4.1.0] hept-1-yl) methanol	-	-	2.26	-
20	1-Decanol,2-octyl-	-	-	2.17	-
21	1-Pentanol, 2-propyl-	-	-	1.37	0.20
22	l-menthol	-	-	-	1.04
23	Cedren-13-ol, 8-	-	-	-	3.87
24	Phytol	-	-	-	1.05
25	Octanal	1.12	-	0.19	0.13
26	(2*E*)-2-Octenal	0.60	-	0.38	-
27	Nonanal	6.48	1.71	1.85	1.44
28	Decanal	1.65	0.88	0.80	0.84
29	Dec-2-enal	1.37	-	-	-
30	(*Z*)-7-Hexadecenal	-	-	-	3.75
31	(+)-(R)-Limonene	1.05	-	0.07	0.08
32	l-Caryophyllene	8.39	13.88	8.36	5.30
33	Farnesene	2.74	11.86	7.05	6.16
34	Ocimene	-	0.87	1.21	-
35	2,5,6-Trimethyl-1,3,6-heptatriene	-	1.73	-	-
36	β-Sesquiphellandrene	-	4.60	2.81	4.24
37	Trans-7-tetradecene	-	1.86	-	-
38	Alloaromadendrene	-	30.37	13.68	10.67
39	1-Chloro-7-heptadecene	-	1.71	-	-
40	2,5-Dimethyl-3-vinyl-1,4-hexadiene	-	1.41	-	-
41	Cedrene	-	-	2.64	2.36
42	4,11,11-Trimethyl-8-methylene-bicyclo [7.2.0] undEc-4-ene	-	-	3.40	-
43	5-Methyl-2-undecene	-	-	1.04	-
44	α-Copaene	-	-	-	2.74
45	α-Caryophyllene	-	-	-	1.23
46	Undecane	0.50	0.73	0.32	0.07
47	Dodecane	0.86	3.58	0.42	0.43
48	Dodecane,4,6-dimethyl	3.55	-	0.59	0.61
49	Tetradecane	2.74	-	3.45	2.59
50	5-Butylnonane	2.72	-	-	-
51	2,6,10-Trimethyldodecane	0.93	1.26	2.40	1.22
52	Heptadecane	1.50	-	2.25	1.32
53	Octadecane	3.80	-	-	-
54	Nonadecane	1.29	-	-	0.42
55	3,8-Dimethyldecane	0.30	0.83	1.62	1.28
56	3-Methylundecane	0.36	-	1.89	-
57	3,7-Dimethylnonane	-	0.54	1.24	-
58	Tridecane	-	1.27	-	-
59	5-Cyclohexylicosane	-	0.98	1.44	1.70
60	2,3,8-Trimethyldecane	-	1.75	0.69	-
61	Hexadecane	-	0.85	1.17	0.70
62	Dodecane, 2-cyclohexyl-	-	0.71	-	1.41
63	2,3,5-Trimethyldecane	-	2.93	-	-
64	Dodecane, 6-methyl-	-	-	1.60	0.64
65	Undecane, 4,4-dimethyl-	-	-	1.04	-
66	7-Cyclohexylicosane	-	-	1.52	-
67	Pentadecane	-	-	2.16	-
68	Heneicosane	-	-	1.02	1.79
69	(1-Propyldecyl) cyclohexane	-	-	0.69	1.05
70	Undecane, 6-methyl-	-	-	1.38	-
71	Undecane, 3-methyl-	-	-	1.82	-
72	Decane, 2,3,5,8-tetramethyl-	-	-	1.76	-
73	Octane, 2,3,3-trimethyl-	-	-	2.20	-
74	Heptadecane, 2,6,10,14-tetramethyl-	-	-	1.37	-
75	Hexane, 2,3,3-trimethyl-	-	-	-	1.27
76	Cyclohexane, 1,5-diethenyl-3-methyl-2-methylene-, (1.alpha.,3.alpha., 5.alpha.) -	-	-	-	1.64
77	5-Methyl-5-propylnonane	-	-	-	1.60
78	Cyclohexane, 1-ethyl-2-propyl-	-	-	-	1.19
79	Nonane, 2-methyl-5-propyl-	-	-	-	2.73
80	Tetradecane, 5-methyl-	-	-	-	1.19
81	Undecane, 3,8-dimethyl-	-	-	-	1.53
82	Tetracontane, 3,5,24-trimethyl-	-	-	-	2.02
83	Tridecane, 5-cyclohexyl-	-	-	-	1.27
84	Tetratetracontane	-	-	-	1.52
85	(1-Propylheptyl) cyclohexane	-	-	-	1.37
86	Eicosane	-	-	-	2.31
87	6-Methyl-6-hepten-2-one	0.76	-	-	-
88	4-Hexen-2-one, 3-methyl-	-	-	0.06	-
89	3-Hexanone	-	-	0.06	-
90	Ethanone, 1-cyclopropyl-	-	-	0.06	-
91	3,5-Dimethyl-4-octanone	-	-	0.13	-
92	Umbellulon	-	-	0.24	0.03
93	5-Hepten-2-one, 6-methyl-	-	-	0.60	0.24
94	1-Cyclopropylethanone	-	0.07	-	-
95	2-Butylfuran	0.30	-	-	-
96	Butanoic anhydride	0.71	0.13	0.06	-
97	Isovaleric anhydride	-	0.16	0.14	-
98	Eicosapentaenoic	-	2.01	-	-
99	1-octoxyoctane	-	0.43	-	0.74
100	2-(7-Heptadecynyloxy) tetrahydro-2*H*-pyran	-	-	2.05	-
101	2,5-Dimethyl pyrazine	0.93	-	-	-
102	Isobutyrophenone	0.51	-	-	-
103	2,4’-Bistetrahydrofuran	-	-	-	0.56

- means not detected. (Abbreviations: TBH—Huantai tartary buckwheat tea, TBC—tartary buckwheat chrysanthemum tea, TBR—tartary buckwheat rose tea, TBJ—tartary buckwheat jasmine tea).

**Table 2:** Total flavonoid content (TFC), total phenolic content (TPC) and antioxidant ability in the tea infusion of tartary buckwheat tea (TBT).

Sample	TFC (mg/g)		TPC (mg/g)		DPPH Free Radical Scavenging Ability/%		•OH Radical Scavenging Ability/%		Total Reducing Power/ (OD Values)	
	FTI	STI	FTI	STI	FTI	STI	FTI	STI	FTI	STI
TBH	7.04 ± 0.10 ^q^	4.62 ± 0.08 ^b^	5.17 ± 0.06 ^s^	4.14 ± 0.01 ^d^	44.94 ± 1.63 ^r^	39.06 ± 2.18 ^c^	43.46 ± 4.32 ^r^	27.78 ± 0.39 ^c^	1.81 ± 0.01 ^r^	1.32 ± 0.04 ^b^
TBC	12.03 ± 0.87 ^p^	8.98 ± 0.84 ^a^	7.84 ± 0.05 ^q^	6.47 ± 0.19 ^b^	71.76 ± 3.99 ^pq^	69.15 ± 5.04 ^a^	85.90 ± 4.58 ^q^	58.11 ± 1.61 ^b^	2.25 ± 0.00 ^p^	2.04 ± 0.02 ^a^
TBR	3.73 ± 0.12 ^r^	3.58 ± 0.26 ^bc^	9.49 ± 0.01 ^p^	9.27 ± 0.09 ^a^	68.04 ± 1.06 ^q^	59.24 ± 2.05 ^b^	97.24 ± 0.30 ^p^	87.72± 0.92 ^a^	2.28 ± 0.03 ^p^	2.16 ± 0.11 ^a^
TBJ	3.92 ± 0.10 ^r^	3.33 ± 0.27 ^c^	5.43 ± 0.01 ^r^	4.58 ± 0.02 ^c^	76.30 ± 1.19 ^p^	62.33 ± 5.71 ^ab^	92.65 ± 1.85 ^pq^	89.46 ± 1.94 ^a^	1.94 ± 0.05 ^q^	1.58 ± 0.20 ^b^

The same letter in the Figures signifies no significant difference, and the letters p–s and a–d indicates the difference of the first tea infusion (FTI) and second tea infusion (STI), respectively (*p* < 0.05) (Abbreviations: TBH—Huantai tartary buckwheat tea, TBC—tartary buckwheat chrysanthemum tea, TBR—tartary buckwheat rose tea, TBJ—tartary buckwheat jasmine tea).

## References

[1] Morishita T., Yamaguchi H., Degi K. (2007). The contribution of polyphenols to antioxidative activity in common buckwheat and Tartary buckwheat grain. Plant Prod. Sci..

[2] Bonafaccia G., Gambelli L., Fabjan N., Kreft I. (2003). Trace elements in flour and bran from common and tartary buckwheat. Food Chem..

[3] Kim S.-J., Zaidul I.S.M., Suzuki T., Mukasa Y., Hashimoto N., Takigawa S., Noda T., Matsuura-Endo C., Yamauchi H. (2008). Comparison of phenolic compositions between common and tartary buckwheat (Fagopyrum) sprouts. Food Chem..

[4] Sun X., Li W., Hu Y., Zhou X., Ji M., Yu D., Fujita K., Tatsumi E., Luan G. (2018). Comparison of pregelatinization methods on physicochemical, functional and structural properties of tartary buckwheat flour and noodle quality. J. Cereal Sci..

[5] Pomeranz Y., Lorenz K. (1983). Buckwheat: Structure, composition, and utilization. C R C Crit. Rev. Food Technol..

[6] Lin R.F., Zhou X.L., Ren G.X., Bian J.S., Fang S. (2005). Production and Trading of Buckwheat in China, Nutrition and Food. Food Sci..

[7] Acquistucci R., Fornal J. (2010). Italian buckwheat (*Fagopyrum esculentum*) starch: Physico-chemical and functional characterization and in vitro digestibility. Food/Nahrung.

[8] Skrabanja V., Liljeberg Elmståhl H.G., Kreft I., Björck I.M. (2001). Nutritional properties of starch in buckwheat products: Studies in vitro and in vivo. J. Agric. Food Chem..

[9] Guo H., Yang X., Zhou H., Luo X., Qin P., Li J., Ren G. (2017). Comparison of Nutritional Composition, Aroma Compounds, and Biological Activities of Two Kinds of Tartary Buckwheat Tea. J. Food Sci..

[10] Wang L., Yang X., Qin P., Shan F., Ren G. (2013). Flavonoid composition, antibacterial and antioxidant properties of tartary buckwheat bran extract. Ind. Crops Prod..

[11] Li S.Q., Zhang Q.H. (2001). Advances in the development of functional foods from buckwheat. C R C Crit. Rev. Food Technol..

[12] Qin P., Li W., Yang Y., Ren G. (2013). Changes in phytochemical compositions, antioxidant and α-glucosidase inhibitory activities during the processing of tartary buckwheat tea. Food Res. Int..

[13] Kreft I., Fabjan N., Yasumoto K. (2006). Rutin content in buckwheat (Fagopyrum esculentum Moench) food materials and products. Food Chem..

[14] Vogrincic M., Timoracka M., Melichacova S. (2010). Degradation of Rutin and Polyphenols during the Preparation of Tartary Buckwheat Bread. J. Agric. Food Chem..

[15] Ali Esmail A.-S. (2018). Pharmacological and therapeutic effects of jasminum sambac-a review. Indo Am. J. Pharm. Sci..

[16] Kaneko S., Chen J., Wu J., Suzuki Y., Ma L., Kumazawa K. (2017). Potent Odorants of Characteristic Floral/Sweet Odor of Chinese Chrysanthemum Flower Tea Infusion. J. Agric. Food Chem..

[17] Vinokur Y., Rodov V., Reznick N., Goldman G., Horev B., Umiel N., Friedman H. (2006). Rose Petal Tea as an Antioxidant-rich Beverage: Cultivar Effects. J. Food Sci..

[18] Peng L.X., Zou L., Wang J.B., Zhao J.L., Xiang D.B., Zhao G. (2015). Flavonoids, Antioxidant Activity and Aroma Compounds Analysis from Different Kinds of Tartary Buckwheat Tea. Indian J. Pharm. Sci..

[19] Qin P., Ma T., Wu L., Shan F., Ren G. (2011). Identification of Tartary Buckwheat Tea Aroma Compounds with Gas Chromatography-Mass Spectrometry. J. Food Sci..

[20] Li C., Xu F., Cao C., Shang M.Y., Zhang C.Y., Yu J., Liu G.X., Wang X., Cai S.Q. (2013). Comparative analysis of two species of Asari Radix et Rhizoma by electronic nose, headspace GC–MS and chemometrics. J. Pharm. Biomed. Anal..

[21] Yang X., Liu Y., Mu L., Wang W., Zhan Q., Luo M., Tian H., Lv C., Li J. (2018). Discriminant research for identifying aromas of non-fermented Pu-erh tea from different storage years using an electronic nose. J. Food Process. Preserv..

[22] Lan Y., Wu J., Wang X., Sun X., Hackman R.M., Li Z., Feng X. (2017). Evaluation of antioxidant capacity and flavor profile change of pomegranate wine during fermentation and aging process. Food Chem..

[23] Yang W., Yu J., Pei F., Mariga A.M., Ma N., Fang Y., Hu Q. (2016). Effect of hot air drying on volatile compounds of Flammulina velutipes detected by HS-SPME-GC-MS and electronic nose. Food Chem..

[24] Qiu S., Gao L., Wang J. (2015). Classification and regression of ELM, LVQ and SVM for E-nose data of strawberry juice. J. Food Eng..

[25] Chen Q., Liu A., Zhao J., Qin O. (2013). Classification of tea category using a portable electronic nose based on an odor imaging sensor array. J. Pharm. Biomed. Anal..

[26] Starowicz M., Koutsidis G., Zieliński H. (2018). Sensory analysis and aroma compounds of buckwheat containing products-a review. Crit. Rev. Food Sci. Nutr..

[27] Qin P., Wang Q., Shan F., Hou Z., Ren G. (2010). Nutritional composition and flavonoids content of flour from different buckwheat cultivars. Int. J. Food Sci. Technol..

[28] Liu Y., Cai C., Yao Y., Xu B. (2019). Alteration of phenolic profiles and antioxidant capacities of common buckwheat and tartary buckwheat produced in China upon thermal processing. J. Sci. Food Agric..

[29] Guo X.-D., Wu C.-S., Ma Y.-J., Parry J., Xu Y.-Y., Liu H., Wang M. (2012). Comparison of milling fractions of tartary buckwheat for their phenolics and antioxidant properties. Food Res. Int..

[30] Fabjan N., Rode J., Kosir I.J., Wang Z., Zhang Z., Kreft I. (2003). Tartary buckwheat (Fagopyrum tataricum Gaertn.) as a source of dietary rutin and quercitrin. J. Agric. Food Chem..

[31] Xu B., Chang S.K.C. (2012). Comparative study on antiproliferation properties and cellular antioxidant activities of commonly consumed food legumes against nine human cancer cell lines. Food Chem..

[32] Ren S.C., Sun J.T. (2014). Changes in phenolic content, phenylalanine ammonia-lyase (PAL) activity, and antioxidant capacity of two buckwheat sprouts in relation to germination. J. Funct. Foods.

[33] Trouillas P., Calliste C.A., Allais D.P., Simon A., Marfak A., Delage C., Duroux J.L. (2003). Antioxidant, anti-inflammatory and antiproliferative properties of sixteen water plant extracts used in the Limousin countryside as herbal teas. Food Chem..

[34] Zielinska D., Szawara-Nowak D., Zielinski H. (2013). Antioxidative and Anti-Glycation Activity of Buckwheat Hull Tea Infusion. Int. J. Food Prop..

[35] Irakli M., Tsifodimou K., Sarrou E., Chatzopoulou P. (2017). Optimization infusions conditions for improving phenolic content and antioxidant activity in Sideritis scardica tea using response surface methodology. J. Appl. Res. Med. Aromat. Plants.

[36] Lipinski B. (2011). Hydroxyl radical and its scavengers in health and disease. Oxid. Med. Cell. Longev..

[37] Fang Y.Z., Yang S., Wu G. (2002). Free radicals, antioxidants, and nutrition. Nutrition.

[38] Wang L., Sun X., Fan L., Dan Y., Liu X., Huang W., Zhan J. (2015). Dynamic changes in phenolic compounds, colour and antioxidant activity of mulberry wine during alcoholic fermentation. J. Funct. Foods.

[39] Lee L.-S., Choi E.-J., Kim C.-H., Sung J.-M., Kim Y.-B., Seo D.-H., Choi H.-W., Choi Y.-S., Kum J.-S., Park J.-D. (2016). Contribution of flavonoids to the antioxidant properties of common and tartary buckwheat. J. Cereal Sci..

[40] Sun T., Ho C.T. (2005). Antioxidant activities of buckwheat extracts. Food Chem..

[41] Torri L., Rinaldi M., Chiavaro E. (2014). Electronic nose evaluation of volatile emission of Chinese teas: From leaves to infusions. Int. J. Food Sci. Technol..

[42] Molinari R., Costantini L., Timperio A.M., Lelli V., Bonafaccia F., Bonafaccia G., Merendino N. (2018). Tartary buckwheat malt as ingredient of gluten-free cookies. J. Cereal Sci..

[43] Bi W., Shen J., Gao Y., He C., Peng Y., Xiao P. (2016). Ku-jin tea ( Acer tataricum subsp. ginnala or A. tataricum subsp. theiferum), an underestimated functional beverage rich in antioxidant phenolics. J. Funct. Foods.

[44] Ghimeray A.K., Sharma P., Phoutaxay P., Salitxay T., Sun H.W., Sang U.P., Park C.H. (2014). Far infrared irradiation alters total polyphenol, total flavonoid, antioxidant property and quercetin production in tartary buckwheat sprout powder. J. Cereal Sci..

[45] Li B., Li Y., Hu Q. (2016). Antioxidant activity of flavonoids from tartary buckwheat bran. Toxicol. Environ. Chem. Rev..

[46] Zhai F.H., Wang Q., Han J.R. (2015). Nutritional components and antioxidant properties of seven kinds of cereals fermented by the basidiomycete Agaricus blazei. J. Cereal Sci..

